# Value configurations for balancing standardization and customization in chronic care: a qualitative study

**DOI:** 10.1186/s12913-021-06844-z

**Published:** 2021-08-21

**Authors:** Christian Colldén, Andreas Hellström, Ida Gremyr

**Affiliations:** 1 Department of Technology Management and Economics, Division of Service Management and Logistics, Chalmers University of Technology, Gothenburg, Sweden; 2 Department of Psychotic Disorders, Sahlgrenska University Hospital, Gothenburg, Sweden

**Keywords:** Healthcare management, Standardization, Customization, Individualization, Personalization, Value configurations, Value in healthcare, Organizational architecture, Mental healthcare, Quality in healthcare

## Abstract

**Background:**

Demands for both customization and standardization are increasing in healthcare. At the same time, resources are scarce, and healthcare managers are urged to improve efficiency. A framework of three *value configurations* – shop, chain, and network – has been proposed for how healthcare operations can be designed and organized for efficient value creation. In this paper, use of value configurations for balancing of standardization and customization is explored in the context of care for chronic mental conditions.

**Methods:**

A typical case is presented to illustrate the manifestations of conflicting demands between customization and standardization, and the potential usefulness of the value configurations framework. Qualitative data were collected from managers and care developers in two focus groups and six semi-structured interviews, completed by a national document describing a care pathway. Data were coded and analysed using an insider-outsider approach.

**Results:**

Operationalization of the balance between standardization and customization were found to be highly delegated and ad hoc. Also, the conflict between the two demands was often seen as aggravated by scarce resources. Value configurations can be fruitful as a means of discussing and redesigning care operations if applied at a suitable level of abstraction. Applied adequately, all three value configurations were recognized in the care operations for the patient group, with shop as the overarching configuration. Some opportunities for improved efficiency were identified, yet all configurations were seen as vital in the chronic care process.

**Conclusions:**

The study challenges the earlier proposed organizational separation of care corresponding to different value configurations. Instead, as dual demand for customization and standardization permeates healthcare, parallel but explicated value configurations may be a path to improved quality and efficiency. Combined and intermediate configurations should also be further investigated.

**Supplementary Information:**

The online version contains supplementary material available at 10.1186/s12913-021-06844-z.

## Background

Two seemingly conflicting demands permeate healthcare, from entire systems to care units, to individual professionals [1, 2]. On the one hand, standardization is promoted to improve efficiency of healthcare systems struggling to limit escalating costs and ensure best practice [3–5]; on the other hand, customization is asked for by both patients and authorities, propagating for shared decision-making [6, 7], patient-centeredness [8], precision medicine [9], and care co-creation [10]. The issue of standardization versus customization is not new, neither in general [11] or to healthcare [1, 4]. In healthcare, the demands are sometimes connected to different logics of managers and professionals [1, 12]. However, the question of how to strike the balance between standardization and customization is often left to individual healthcare managers and professionals.

### Choosing or combining customization and standardization?

Simply choosing between standardization and customization is not feasible, since the dual nature of quality containing both objective and subjective elements requires the coexistence of both standardization and customization [13]. Generally, customization can be seen as emphasizing quality defined as patient outcomes and experiences, while standardization to a greater extent emphasize efficiency and puts quality in relation to costs. However, standardization and customization are multifaceted concepts and Mannion and Exworthy [1] outline four subtypes for each of the two demands, which all are present in healthcare. In this paper, we focus primarily on standardization as “procedural standards” and customization as “personalization”. Applying these views, standardization focuses more on compliance to best practices and evidence-based medicine as one dimension of quality, while customization values the unique properties and desires of the individual patient as another dimension of quality. Whereas a standardized “one size fits all” approach may ensure cost-efficiency, service reliability, and decreased number of mistakes [13], customization improves the probability of meeting patients’ needs and enhancing the patient’s experience and engagement. An increasing trend in healthcare organizations is to adopt standards of care [14]. However, standardized solutions might have negative consequences in healthcare [15], and several studies suggest that an increased customization might magnify the value provided to customers [16–18].

Moreover, in contrast to the view of conflicting demands there are scholars that see standardization and customization in healthcare as complementary [11, 19, 20], or as two extremes on a continuum of strategies [21]. Inspired by the industry-originated concept of *mass customization* [11, 22] there has been an increasing interest in *customized care* [20]. Customized care aims to allow increased individualization and personalization in healthcare at affordable cost levels, maintaining the efficiency and economies of scale that standardization promises [23]. However, a common strategy for organizing and managing for care customization is still lacking [19]. Some initial propositions for operationalizations aimed to improve care efficiency have been presented, for example by discrete choice-models [24], use of multidisciplinary teams [25–28], and care modules [29, 30]. However, more guidance for managers on how to apply and organize for customization and standardization is still needed. A core challenge is to identify what activities that correspond to standardization and customization [23] and, furthermore, how to organize effectively for these different activities in practice.

### The value configurations framework

Addressing the issue of practically organising for standardization and customization, Fjeldstad et al. [31] have suggested three different value configurations as models for how patient value is created: shop, chain, and network. These value configurations can be used to illustrate how different activities are carried out, but also how competences, services, responsibilities, and level of standardization are organized to fulfill the needs of the patients. Principally, *shop* implies customized problem-solving, *chain* entails linked care processes with little variation, and *network* focuses on facilitating care and support from different actors in a system over time based on the needs of a specific patient. In terms of cost effectiveness, shops provide tailored but expensive solutions to unique problems; chains provide standard solutions that are cheaper than shops per unit and that benefit from larger scales; while in networks, value is created jointly and flexibly by its actors at an even lower cost per unit and significant scale advantages [32]. Each configuration implies different roles for patients, professionals, managers, and technology. For example, in shops, healthcare professionals are experts with the liberty to design care for the individual patient, whereas in networks, patients and external actors are experts and healthcare professionals are facilitators of value creation [31].

To utilize the full potential for cost-efficient value creation, Hwang and Christensen [33] argued that activities corresponding to different value configurations should be organizationally separated. Their view was that today’s healthcare mainly leans towards customization in ’disjointed‘ shop configurations but argued that care efficiency could be greatly improved if care could be organized as networks, chains, or shops (in descending order of preference) instead.

In the pursuit of guidance on how to efficiently manage and organize care while balancing demands for customization and standardization, we choose to focus on the value configurations framework, as it provides practically oriented guidance for how to organize care to be more cost-efficient.

### The case of chronic mental care

Increased cost-efficiency is a well-known target for healthcare providers in general. This is particularly relevant for chronic care, as medical development has entailed increasing volumes of patients living with long-term and/or multiple conditions [34, 35]. Further, mental disabilities add considerably to this cost development [36]. One example of a chronic mental conditions is schizophrenia, which is characterized by great complexity of care, and high costs over a long period of time [37]. The patients often suffer from cognitive impairments and hence have particular and highly individual difficulties functioning as independent actors in relation to healthcare. Also, care is often provided for many decades from an onset in early adulthood and by several caregivers, from healthcare, municipalities, and family. These characteristics are evident in many conditions but are especially pronounced in chronic mental conditions which have, therefore, been purposefully selected [38] as an illustrative case for this study.

### Purpose and research questions

The purpose of this study is to explore how value configurations can be used to organizationally balance demands for customization and standardization in the quest for cost-efficient care. First, we explore manifestations of conflicting demands and the potential in applying the value configurations framework in a care setting for chronic mental conditions. We then discuss how the conflicting demands for standardization and customization can be managed organizationally in practice. The study is guided by two research questions:

RQ1: How are the demands for customization and standardization manifested in managerial practice?

RQ2: How can the value configurations framework support the balancing of customization and standardization in practice?

## Method

### Research design and setting

As the purpose of this paper is of an explorative nature, it requires a proximity to the phenomenon studied, and hence a qualitative study of a specific case is suitable [39]. The study was conducted in a department within the Sahlgrenska University Hospital, Sweden, providing care for patients with chronic psychotic disorders (diagnosed F20.0–F29.9 in the International Classification of Diseases [40]). The department includes both hospital-based in-patient units and out-patient units located externally. The case sampling was purposive, focusing one typical case [38] of care for patients with a chronic disease characterized by episodes, for example acute episodes and continuous, monitoring episodes. Hence, the case is purposive in illustrating a variety of care episodes with different types of demands on customization and standardization within the same organizational unit.

### Data collection and analysis

Departing from the purpose, data were collected in two steps: focus groups (with nine participants) to spur discussions around value configurations and semi-structured interviews with six interviewees to gain insights on the focus group data as well as on value configurations as a means of handling tensions inherent in customization and standardization.

First, two focus groups with managers and care developers (n = 9) were conducted to capture their views on the potential for using value configurations as a concept and the dynamics in the discussions [41]. In the focus groups, the three value configurations[31, 32] were presented, followed by written individual reflections about value configurations in relation to the department. Thereafter, participants worked in pairs to identify parts of the care operations corresponding to any of the value configurations. Finally, the entire group discussed how the operations could be described in terms of value configurations and potential concurrency or transitions between configurations. For the analysis, the focus group discussions were transcribed verbatim and coded using NVivo 12. Data were first deductively coded by theoretically derived themes based on Fjeldstad et al. [31], followed by inductive coding to identify new codes capturing emerging concepts [42]. In this case, inductive codes were developed to capture the focus group discussions on challenges in, for example, defining and separating different value configurations (for coding scheme, see Additional file 1).

Moreover, evidence from secondary data was added in the analysis of the focus groups by including a national care pathway for patients recently diagnosed with schizophrenia (in this paper referred to as “the care pathway”). The care pathway was initiated by the government and developed by a multidisciplinary expert group. At the time of the focus groups, it was referred to care providers for review. The care pathway was included in the NVivo file and analysed through the lens of standardization and customization.

Second, semi-structured interviews (*n* = 6) were conducted with interviewees selected based on a key informant sampling [43]. All but two interviewees had also participated in the focus groups, and all held positions as unit managers in the department of study. All but one of the interviewees were managing out-patient units. Selection of interviewees was based on a comprehensive sampling [44] inviting all unit managers for care units within the department; however, one unit manager was excluded as she had less than half a year experience of management. Based on the purpose, a sampling criterion was management experience from psychiatry. To have insights in case-specific organizational challenges a minimum of six month in a relevant position was required for study participation. A majority of the unit managers within the department were either interviewed or participated in the focus groups. To ensure that quotes cannot be connected to specific individuals, no further attributes of the interviewees will be described, and quotes will be referred to only by interview number (I1-6).

The interviews had two main purposes: enhancing the validity of the focus group findings and providing insights to the management of standardization and customization. In the first part of the interviews, the discussions from the focus groups on value configurations were revisited, providing additional input and data as well as enhancing quality by respondent validity [45]. The second part of the interviews focused on insights to standardization and customization, with interview questions such as “In what ways do these demands [for customization and standardization] manifest at your unit?”, “What challenges do the demands involve for you as manager?” and “Can you see parts of your operation that could be separated [by value configuration] to a higher degree than today?” The interviews lasted between 41 and 56 minutes and the answers were transcribed verbatim and coded using NVivo 12 (for coding scheme, see Additional file 2).

### Research quality

Overall, multiple sources of data and multiple investigators were used to triangulate data and enhance the findings [41, 46]. Written informed consent was obtained from all participants and no patient data was used. Primary data were collected from focus groups and interviews, and secondary data from the care pathway. In terms of multiple investigators, the authors took on different roles in different parts of the research study; for example, the focus groups and subsequent analyses were conducted jointly by the first and second authors, applying an insider-outsider approach [47, 48] with the first author being an insider with ten years of experience from both clinical and managerial work at the department studied. To gain complimentary insights and enhance confidence in the results, the first and third authors jointly analysed the interview data [49]. To further enhance validity of our findings, coding schemes were developed including both deductively and inductively derived codes (Additional files 1 and 2), as a means of providing a clear description and trail of the data analysis [45].

## Results

This section describes the results in three parts emanating from the two focus groups, the care pathway, and the interviews.

### Conflicting demands

The parallel and conflicting demands for customization and standardization were recognized by all interviewees and also evident in the care pathway. The explicit rationale for introducing care pathways to Swedish healthcare is to support implementation of best practice (i.e. standardization), but even in the title, it is stated to be a “person-centred care pathway”, hence implying customization as a key demand. Parallel demands for standardization and customization also permeated the content of the care pathway document. For example, some guidelines allowed for a high degree of customization (e.g. “Adapted reception of patients”), while others specified detailed descriptions of interventions and content of certain visits (e.g. what questions to ask about lifestyle and habits).

At an operational level, the first-line managers spoke from experience that in addition to demands for standardization and customization, the scarcity of resources exerted a pressure to increase efficiency or prioritize care. This economic pressure accentuates the need for a balance between the demands for standardization and customization even more. As stated by one interviewee, “The hard part is to get demands for cost-cuts or improved efficiency at the same time as I shall follow the rules for good care and … the patients should have a care that is as individualized as possible” (I3). Opinions diverged about which demand was the most problematic in relation to scarce resources, and two interpretations of standardization crystallized. First, standardization was seen as a demand to offer a broader range of investigations and interventions to all patients as a ‘floor level’/minimum, hence driving costs. One interviewee said that “requirements for increased efficiency … is a contradictory demand to that we are obliged to conduct yearly physical examinations for all [patients]” (I6). Second, standardization was also seen as a means to rationalize care, putting a ‘roof’/maximum on what care each patient can be expected to get (e.g. number of visits or access to certain interventions). For example, one interviewee was of the opinion that “when there are demands for efficiency and standardization, then it is easier to say, ‘We have this way of working, this is what we are offering’ … there is an efficiency in that of course” (I6).

However, irrespective of the interpretation of standardization, it was perceived as being in conflict with the demand for customization, which was seen as “something that many of [the clinicians] are passionate about [while] standardization is perceived as something that is forced upon the operations … from politicians and top management” (I5). Standardization, as a ‘roof’, directly limits the professional freedom to tailor care for the patients with the greatest needs, while standardization, as a ‘floor’, allows additional care tailored to the individual, but the scarce resources considerably limit the room for such additions. For example, one manager said that “Of course there are discussions [about standardization versus customization] from time to time, but generally the discussion is rather ‘If we are to do all this every year, then give us the resources for it’” (I5).

When asked about how they manage these conflicting demands, the interviewed managers described the handling as ad hoc and delegated. No one presented any structured tool or process for how to resolve or control the balancing of standardization and customization. Instead, they described how the issue is handled by individual clinicians or by interdisciplinary teams. The managers described themselves as available for support to their employees, but seldom experienced that the professionals turned to them for help. Rather, forums for conversation and sense-making in the interdisciplinary teams were emphasized as important ways to handle these matters.

### Value configurations

Most participants had no problem finding examples of the three different configurations for value creation in the operations of the department. A recurring perception was that interdisciplinary teamwork is the core of the out-patient care operations, corresponding to a shop configuration. Diagnostic investigation and care during acute relapses were mostly considered a chain configuration, but some participants argued that the diagnostic investigation could be seen as a shop, depending on the level of abstraction. Similarly, in-patient care was described either as corresponding to a shop or a chain configuration. Care for patients in stable phases was seen to have many elements of network configuration, with, however, a unique network for each patient.

 The participants sometimes struggled to find the most useful level of abstraction to describe operations. For example, at an overall level, a hospitalization period follows a standard course, but studied in more detail, an individual patient journey is subject to many individualized decisions by interdisciplinary teams and can differ in both content and length. Also, standards for a specific investigation process and individual plans in cases of relapse are examples that could be seen as either structured parts of a shop or separate elements configured as chains. As one participant stated, “A shop is a collection of chains” (manager participating in a focus group).

**Fig. 1:**
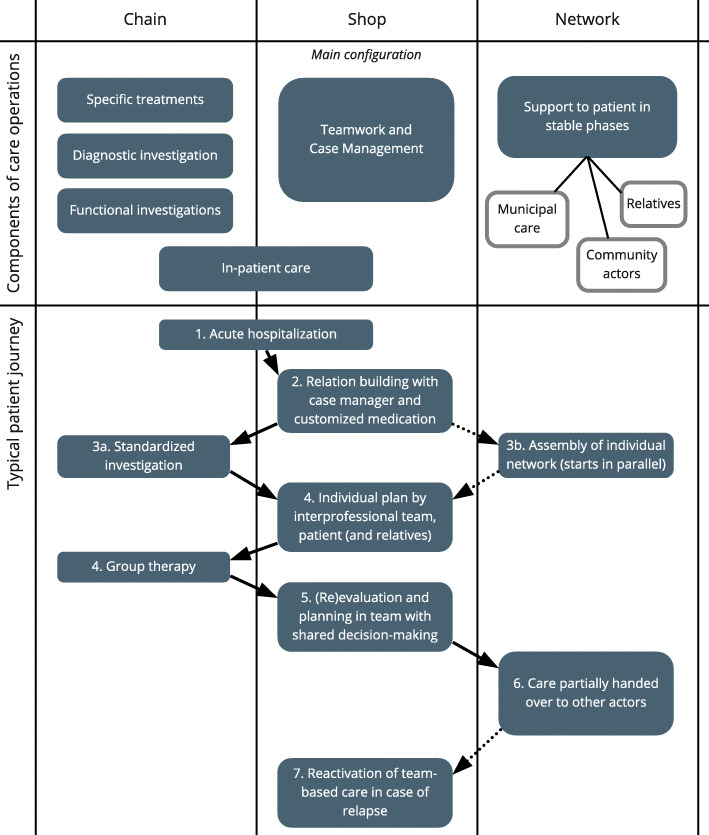
The main care operations of the department by corresponding value configuration. Types of operational activities as mapped by the focus groups and a schematic description of a typical patient journey in the studied department.

In the focus groups, the participants jointly developed a description of the care operations at the department following the conceptual descriptions of the three value configurations. As illustrated in Fig. 1, shop was seen as the overall configuration, with interdisciplinary teams and case management at the centre of out-patient care. Within this configuration, the patient can undergo standardized processes or only receive customized care in a shop depending on individualized decisions of the teams taken together with patients. When in a stable phase, the care process moves towards a more network-based configuration, facilitating support by individually designed networks of private and professional actors. As stated by an interviewee, “The further you come in the process, the more the needs of social character crystallize – housing support in the patient’s home and so on – then you have to attribute the network to a greater extent” (I4). Other examples of actors that can be included in the patients’ networks are municipal home care, employment service, assisted living facilities, custodians, family members and friends. These actors have different roles, but in different ways they all support the patient by helping to avoid relapses and improving their quality of life.

In-patient care was seen as a mixture of shop and chain configurations: shop in terms of individually tailored medication and planning, and chain because of the overall trajectory with admission, information, treatment, planning and discharge, with elements of standardized routines. The national care pathway also had traits of several value configurations. At a general level, the pathway is described as a chain of large segments, where each segment consists of chunks of guidelines to use when applicable. As exemplified above, the guidelines ranged from advising customization (corresponding to shop configuration) to specifying in-depth the steps of certain interventions (as delimited chains).

### Managing conflicting demands by use of value configurations

Both the focus group participants and the interviewees concluded that value configurations can be fruitful as a base for discussion and (re)design of operations. However, the feasibility of separating care corresponding to different value configurations organizationally was questioned. Instead, it was argued that the unpredictability of the mental condition and the importance of the relationship between the clinician and the patient required integration, or parallelism of configurations. As argued in one of the focus groups, “The network should almost always be a parallel process, because if you are admitted [and have] a custodian, some relatives, and so, they are – if the patient accepts it of course – connected even in the acute phase” (manager participating in a focus group).

Thus, organizational separation of configurations was questioned, except for very limited elements or activities. Nonetheless, the interviewees perceived value configurations useful as a means to concurrently meet demands for customization and standardization. An interviewee illustrates this by saying,

It is interesting to think that we have all these three [value configurations]. I see it very clearly with the flowchart that we work with, from referral to the point where the patient is independent and in a good mental state. And in [the shop] is the team and in [the network] are resource groups and the network around the patient. So that captures … that this is what we are dealing with."(I6)

Furthermore, even though parallelism of configurations was seen as necessary, there were interviewees pointing to the potential in value configurations as a means to develop the care operations, with one of them saying, “I think that to have the network as a target image, is something to strive towards, [but] what we actually do here, that is probably more of a shop” (I4).

The care pathway was only superficially known to the interviewees and the implementation of it had not started yet. When referred to by the managers and developers, it was mentioned mainly as a demand for standardization. Studied in more detail, the care pathway can be interpreted as a general description of a chain but with rough steps. The steps can be seen as “toolboxes” of standardized interventions and guidelines to be used within a shop configuration. However, the “tools” can sometimes be contradictory, as some emphasize customization and others standardization. In the end, it is still left to the team or the care professional to customize what standardized interventions to use in the specific case.

## Discussion

As will be elaborated further in this section, the contributions of this paper are threefold. First, the study provides a hands-on illustration of the conflicting demands of customization and standardization but challenges the connection to different professions or actors [1]. Instead, it is suggested that these demands permeate the system without direct connection to specific actors, logics or hierarchical levels. The study also illustrates how the emphasis on increased efficiency makes the balancing of demands even harder. Second, the framework of value configurations [31, 32] is shown to be recognizable by managers in their practice; however, they argue that it is essential to define the level of abstraction at which the configurations are to be applied. Third, organizational separation of value configurations [33] is contested. Instead, parallelism of configurations is suggested to have a value in itself and a potential way to overcome the conflicting demands of customization and standardization accentuated under the pressure of scarce resources.

In Table 1 the results are summarized corresponding to the research questions. The main contributions and implications for each research question are indicated in the table and further elaborated below.

**Table 1:** Summary of results and contributions in relation to the research questions

Research question	Results	Contributions and implications
RQ1: How are the demands for customization and standardization manifested in managerial practice?	Manifestations of demands: Customization: - Desire for professional freedom - Patient demands - Expressed in some guidelines. Standardization: - Care pathways - Guidelines for evidence-based treatments - Routines for standard follow-ups Standardization has two opposite rationales: - Ensuring best practice (‘roof’) - Limiting and streamlining care (‘floor’) Combining standardization and customization is possible but difficult when resources are becoming ever scarcer.	Demands for standardization and customization permeate the healthcare system without clear connection to specific actors. The clinical pathway and existing guidelines call for parallel handling of the demands, supporting the view that combined strategies are needed [19, 21]. The two rationales for standardization may be related to different attitudes to the demand [23, 50, 51] and deserves further investigation.
RQ2: How can the value configurations framework support the balancing of customization and standardization in practice?	Care operations could be mapped by corresponding value configurations. Disagreements on corresponding value configuration could generally be referred to different levels of abstraction. Care operations can seldom be organizationally separated to strictly follow pure value configurations. The study supported generation of ideas for how to develop care operations to be more cost-efficient.	The value configurations framework [31, 51] may be useful for managers as a lens to understand and redesign organization of care. A common and relevant level of abstraction need to be defined. More research is needed on how different configurations on different hierarchical levels can (or cannot) be combined. Organizational separation of value configurations [33, 51] is not supported. Instead, parallel, combined, and/or intermediate configurations can be a more feasible path to improved efficiency. Some adjacent theories are pointed to for inspiration and future studies.

### RQ1: How are the demands for customization and standardization manifested in managerial practice?

First, it is evident, both from the results of this study and from earlier research [1, 2, 13, 23] that conflicting demands for standardization and customization permeate healthcare. However, the results indicate that these demands are not connected to specific actors or professions, or to different professional or administrative logics, but exist throughout the system, refuting a strict connection to the logics of managerialism and professionalism [1, 12]. The interviewed managers express understanding for both demands and see standardization as useful as a backbone and starting point from which customization can be applied. Also, the care pathway was developed by different care professionals and includes recommendations for both highly standardized procedures and customization to each patient’s needs, without connection to which profession usually conducts the task. Hence, the results support the view that healthcare systems need to balance and combine these demands [21] to provide “the best of two worlds” [11, 20, 52].

However, for managers, customization is essentially something that is delegated to the clinicians, while standardization can be used more explicitly in management. The different views of standardization as a ‘floor’ or a ‘roof’ show the implications that standards can have in communicating and discussing management choices, and may also be a reason for the different attitudes towards standardization that are recognized in literature [23, 50, 51]. The options of what to offer patients are made constantly, mostly by individual clinicians or teams and constrained by available time and resources. Introducing standards can be seen as a help for clinicians, releasing them from the responsibility of rationalizing care, but it can also be resisted by clinicians who would rather make those choices individually, by customization. This discussion is continuously shadowed by the pressure of limited resources, regardless of which hierarchical level they are balanced on. That is, a standard as starting point for best-practice care can be combined with customized additions or alterations. But when time and money are scarce, adding or adapting something for one patient in practice entails that the standard cannot be adhered to for another patient.

### RQ2: How can the value configurations framework support the balancing of customization and standardization in practice?

Value configurations are proposed as a means of managing the limited resources more efficiently based on diversion of care processes and their different nature in terms of standardization and customization [32, 33, 51]. The results show that shop is the predominant value configuration in the setting, as is often the case in healthcare [31]. Chain configuration is recognized but seldom for more than fragments of the operations, while networks are acknowledged as a useful principle that could be further exploited.

The word ‘network’ was established in the daily operations of the studied case and included both parts of the own organization (the ‘case manager’ and the out-patient team), external actors (e.g. municipal home care, assisted living facilities, and custodians), and related private parties (family members and friends). Value creation by use of these networks can be seen to correspond both to a shop configuration, where a support system is tailored by external cooperation, and to a “real” network configuration, where connectivity and value-crating relationships among actors is facilitated and the patient “owns” its problem and resources and co-creates value [31]. Using a strict view of user-networks [33], the case does not correspond to such a model, but the results indicate that utilizing more properties of network configuration in a ‘hybrid configuration’ can still entail increased cost-efficiency for the organization. However, it is important to recognize the effects for *all* actors in these networks, to ensure cost-efficiency from a systems perspective.

Generally, the results suggest that all configurations are inevitably tangled and parallel, in contrast to often advocated distinct presentations [51]. However, the results confirm earlier research proposing that value configurations can be useful on a managerial level to understand and develop care operations [13, 31]. In this endeavour, it is important to apply the framework at a useful level of abstraction. If applied at an overly detailed or general level, most activities can be seen as chain configurations. Based on the results of this case, the most useful level of application appears to be on the managerial level at which care is organized for larger groups of patients with similar conditions.

Applied at the level of first- and second-line management, the results indicate that for chronic mental care, organizational separation of parallel value configurations is not feasible [33]. Two key reasons are suggested. First, the unpredictability of the condition and its effects on cognition entails that the care provider cannot rely on patients to continue to be independent and manage their care contacts over time. Thereby, extended chain processes or pure network configurations are disqualified. Second, the personal relation between patient and clinician is critical. For patients to be able to participate in investigations and interventions, a trustful relationship has to be established. Therefore, too many clinicians cannot be involved directly with the same patient; hence, all clinicians must to some extent be generalists and involved in care corresponding to different value configurations.

These practical examples support the notion that demands for standardization and customization must be handled in combination [19] and as a continuum [11, 21]. They also suggest that even though the value configurations framework cannot be used as a blueprint for organization of distinct types of care operations, value configurations can be one response to the call for more guidance on what activities that lend themselves to standardization and not [23].

While organizationally separated configurations might save time and money in theory [33], we suggest that parallelism of configurations sometimes has a value in itself. In practice, when patients are not able to benefit from care configured in a certain way, they may later need costly hospitalizations. For the studied patient group, parallel configurations, with shop as the overall configuration, might be the most efficient organization. Such a model can also be an approach to further “individualized standardization” [28] and balanced strategies of customized care [21] and mass customization [19, 22].

Furthermore, the potential of standardization [4] is not excluded by the limited appreciation of chain configuration. The care pathway includes elements corresponding to chain configuration at an overall level, but on an operational level it rather supports a traditional shop configuration promoting person-centeredness [8] and shared decision-making [7] but with standardization of some components by clinical guidelines [14]. Hence, the care pathway is a potentially helpful tool to define best practice but does not resolve a conflict of standardization versus customization [52, 53].

The study has several practical implications for healthcare managers. First, it is evident that demands for standardization and customization effect healthcare managers and clinicians. The effects are exerted regardless of organizational strategy, but without deliberate choices about how to balance or combine these demands there is a risk of moral distress among the clinicians [52, 53]. Therefore, managers ought to initiate more explicit conversations – and deliberate choices – about the balancing of standardization and customization in the local setting. Here, the value configurations framework can be used as a starting point for a dialogue. Second, the value configurations framework can help managers in understanding complexity and cost drivers [31]. The framework could become a means to organize care activities amenable to either standardization or customization in a comprehensive way [2, 52] and at the same time improve efficiency [31, 32]. For example, network configuration entails improved efficiency over shop configuration and still allows for person-centeredness and customization. However, it is important to recognize the weaknesses of the value configurations network discussed above, and the need to take inspiration from other, complementary frameworks too.

### Limitations and future research

This case of a chronic mental condition is characterized by long-term and complex care for a patient group with varying individual abilities and attributes. These are specific characteristics, and this study is a single case study, which limits the transferability of the findings. On the other hand, the characteristics are common in many of the growing chronic conditions in healthcare today [34, 35]. Hence, the case provides important insights into principles of how value configurations can be applied as a lens to understand and improve this type of care. Moreover, the study has not sought to compare the usefulness of different existing frameworks aimed to improve value creation and balance standardization and customization in healthcare [4, 13, 20, 53]. Hence, even though we suggest that value configurations can be useful to stimulate dialogue and development, other frameworks applied in a similar context could be an area of future research.

The value configurations framework was chosen because of its practice-oriented descriptions and claimed potential for disruptive improvement of cost-efficiency. The study indicates that it has practical usefulness but challenges its potential as a blueprint for easy-fetched efficiency improvements [33, 51]. The framework needs further development and investigation. This paper suggests that in this endeavour, design of parallel, combined, and/or intermediate configurations should be considered as a complement to separation or refinement of single configurations. Inspiration can be drawn from mass customization [11, 22] and customized care [20, 21]. Also, the results imply that standardization is often manifested as delimited elements, to be combined into a customized care package. This scenario bears a resemblance to service modularity [29, 54, 55], which can also be an interesting theory to inspire future studies.

The level of abstraction at which the value configurations framework is useful have been discussed and can be studied further. The results indicate that care can correspond to one value configuration at a higher hierarchical level, and another at a lower level. These relations and possible combinations are an issue for future studies that may be informed by the concept of ‘hierarchization’ of competing logics [56].

Finally, different value configurations imply different roles and principles for both managers and employees. Balancing of parallel, conflicting demands has noticeable similarities with the balancing of exploration and exploitation, as is the focus in studies of organizational ambidexterity [57]. If organizational separation is refuted, the notion of contextual ambidexterity [58, 59] could provide lessons to be learnt for management practice.

## Conclusions

A need to simultaneously balance demands for customization and standardization under pressure of scarce resources is inherent in healthcare management. The pressure to improve efficiency is growing as medical developments outpace economic reimbursements and demands for standardization and customization increase. In this paper, the framework of value configurations [31, 32, 51] is applied as a means to understand and manage standardization and customization, and at the same time improve the efficient usage of resources. The results confirm the relevance of the issue in practice and the utility of value configurations as a framework for dialogue and operational development. However, to be useful, the framework needs to be applied at a managerial level comprising care for larger groups of patients with similar conditions. Also, this paper questions the solution to separately organize care corresponding to different value configurations [33]. Instead, we suggest that applying parallel but explicated value configurations can sometimes be more feasible. We also point to the need for further development of the value configurations framework to include combined and intermediate configurations. This is especially relevant for medical conditions that require long-term care and impair the patient’s ability to be independent in relation to care. This study, while limited to a single case, provides an insight into managerial challenges on a practical level and illustrates what a practical application of value configurations can look like.

The framework of value configurations and its practical application need further investigation. Both deeper and more action-oriented studies and studies of other care settings and conditions are suggested to provide guidance for how the framework can be of practical and theoretical use. We also point to some theories that may fertilize further development, understanding, and applications of the framework.

## Supplementary Information

**Additional file 1:** Coding schemes for focus groups with illustrative examples

**Additional file 2:** Coding schemes for interviews with illustrative examples

## Data Availability

Coding schemes with illustrative examples supporting the conclusions of this article are included in two additional files: 1. Additional file 1.pdf: Coding schemes for focus groups with illustrative examples 2. Additional file 2.pdf: Coding schemes for interviews with illustrative examples The full transcripts from the focus groups and interviews are available in Swedish from the corresponding author on a reasonable request.

## References

[1] Mannion R, Exworthy M. (Re) Making the procrustean bed? Standardization and customization as competing logics in healthcare. International Journal of Health Policy and Management [Internet]. 2017;6:301–4. Available from: 10.15171/ijhpm.2017.35 10.15171/ijhpm.2017.35 PMC5458790 28812821

[2] Mannion R, Exworthy M. Researching the co-existence and continuity of standardization and customization in healthcare: A response to recent commentaries. International Journal of Health Policy and Management [Internet]. 2018;7:572–3. Available from: 10.15171/ijhpm.2018.07 10.15171/ijhpm.2018.07 PMC6015515 29935138

[3] Andersen H (2015). Enablers for change: A mixed-methods study of Lean-based quality improvement in hospitals.

[4] Lefton R (2008). Reducing variation in healthcare delivery. Healthcare Financial Management.

[5] Pollitt C, Dan S. The impacts of the new public management in Europe: a meta-analysis. COCOPS working paper no. 3. Rotterdam; 2011.

[6] Elwyn G, Laitner S, Coulter A, Walker E, Watson P, Thomson R (2010). Implementing shared decision making in the NHS. British Medical Journal.

[7] Stiggelbout AM, Weijden TV d., Wit MPTD, Frosch D, Legare F, Montori VM, et al. Shared decision making: really putting patients at the centre of healthcare. BMJ [Internet]. 2012;344:e256. Available from: http://www.bmj.com/cgi/doi/10.1136/bmj.e256 10.1136/bmj.e256 22286508

[8] Richards T, Coulter a., Wicks P. Time to deliver patient centred care. Bmj [Internet]. 2015;350:h530–h530. Available from: http://www.bmj.com/cgi/doi/10.1136/bmj.h530 10.1136/bmj.h530 25670197

[9] Denny JC, Collins FS (2021). Precision medicine in 2030 – seven ways to transform healthcare. Cell. Elsevier B.V..

[10] Hardyman W, Daunt KL, Kitchener M. Value Co-Creation through Patient Engagement in Health Care: A micro-level approach and research agenda. Public Management Review [Internet]. 2014;17:1–18. Available from: http://www.tandfonline.com/doi/abs/10.1080/10.1080/14719037.2014.881539

[11] Lampel J, Mintzberg H. Customizing Customization. Sloan Management Review [Internet]. 1996;38:21–30. Available from: https://www.researchgate.net/publication/40962226

[12] Saks M. Competing logics and healthcare: Comment on “(Re) making the procrustean bed? standardization and customization as competing logics in healthcare.” International Journal of Health Policy and Management [Internet]. 2018;7:359–61. Available from: 10.15171/ijhpm.2017.100 10.15171/ijhpm.2017.100 PMC5949229 29626406

[13] Bohmer RMJ (2009). Designing Care: Aligning the Nature and Management of Health Care.

[14] Andritsos DA, Tang CS. Linking process quality and resource usage: An empirical analysis. Production and Operations Management. Wiley-Blackwell; 2014;23:2163–77.

[15] Catena R, Dopson S, Holweg M. On the tension between standardized and customized policies in health care: The case of length-of-stay reduction. Journal of Operations Management. John Wiley and Sons Inc.; 2020;66:135–50.

[16] Fogliatto FS, da Silveira GJC, Borenstein D. The mass customization decade: An updated review of the literature. International Journal of Production Economics. 2012. p. 14–25.

[17] Piller FT, Moeslein K, Stotko CM (2004). Does mass customization pay? An economic approach to evaluate customer integration. Production Planning and Control.

[18] Squire B, Readman J, Brown S, Bessant J (2004). Mass customization: The key to customer value?. Production Planning and Control..

[19] Greenfield D, Eljiz K, Butler-Henderson K (2018). It takes two to tango: Customization and standardization as colluding logics in healthcare: Comment on “(re) making the procrustean bed standardization and customization as competing logics in healthcare. International Journal of Health Policy and Management.

[20] Minvielle E, Fourcade A, Ricketts T, Waelli M. Current developments in delivering customized care: a scoping review. BMC Health Services Research [Internet]. 2021;21:575. Available from: https://bmchealthservres.biomedcentral.com/articles/10.1186/s12913-021-06576-0 10.1186/s12913-021-06576-0 PMC8201906 34120603

[21] Minvielle E, Waelli M, Sicotte C, Kimberly JR (2014). Managing customization in health care: A framework derived from the services sector literature. Health Policy. Elsevier Ireland Ltd.

[22] Fogliatto FS, da Silveira GJC, Borenstein D. The mass customization decade: An updated review of the literature. International Journal of Production Economics [Internet]. Elsevier; 2012;138:14–25. Available from: 10.1016/j.ijpe.2012.03.002

[23] Minvielle E, Sicotte C (2021). The quest of rationality: Standardization in the Delivery of Care. Journal of Organizational Psychology.

[24] Benning TM, Kimman ML, Dirksen CD, Boersma LJ, Dellaert BGC (2012). Combining individual-level discrete choice experiment estimates and costs to inform health care management decisions about customized care: The case of follow-up strategies after breast cancer treatment. Value in Health.

[25] von Dadelszen P, Magee LA, Payne BA, Dunsmuir DT, Drebit S, Dumont GA, et al. Moving beyond silos: How do we provide distributed personalized medicine to pregnant women everywhere at scale? Insights from PRE-EMPT. International Journal of Gynecology and Obstetrics. Elsevier Ireland Ltd; 2015;131:S10–5. 10.1016/j.ijgo.2015.02.008 26433496

[26] Mercer T, Bae J, Kipnes J, Velazquez M, Thomas S, Setji N. The highest utilizers of care: Individualized care plans to coordinate care, improve healthcare service utilization, and reduce costs at an academic tertiary care center. Journal of Hospital Medicine. John Wiley and Sons Inc.; 2015;10:419–24. 10.1002/jhm.2351 25854685

[27] Estape EA, Mays MH, Sternke EA. Translation in Data Mining to Advance Personalized Medicine for Health Equity. Intelligent Information Management. Scientific Research Publishing, Inc,; 2016;08:9–16. 10.4236/iim.2016.81002 PMC4868394 27195185

[28] Ansmann L, Pfaff H. Providers and patients caught between standardization and individualization: Individualized standardization as a solution: Comment on “(re) making the procrustean bed? standardization and customization as competing logics in healthcare.” International Journal of Health Policy and Management [Internet]. 2018;7:349–52. Available from: 10.15171/ijhpm.2017.95 10.15171/ijhpm.2017.95 PMC5949226 29626403

[29] de Blok C, Luijkx K, Meijboom B, Schols J (2010). Modular care and service packages for independently living elderly. International Journal of Operations and Production Management.

[30] Peters V, Vähätalo M, Meijboom B, Barendregt A, Bok L, de Vries E. Elaborating on modular interfaces in multi-provider contexts. International Journal of Operations and Production Management. Emerald Group Holdings Ltd.; 2020;40:1397–419.

[31] Fjeldstad ØD, Johnson JK, Margolis PA, Seid M, Höglund P, Batalden PB. Networked health care: Rethinking value creation in learning health care systems. Learning Health Systems. 2019;1–9. 10.1002/lrh2.10212 PMC7156860 32313837

[32] Stabell CB, Fjeldstad ØD (1998). Configuring Value for Competitive Advantage: On Chains, Shops, and Networks. Strategic Management Journal.

[33] Hwang J, Christensen CM (2008). Disruptive innovation in health care delivery: a framework for business-model innovation. Health affairs (Project Hope).

[34] McPhail SM (2016). Multimorbidity in chronic disease: Impact on health care resources and costs. Risk Management and Healthcare Policy.

[35] Zhao L, Anderson K, Riley G (2018). Association between persistent high costs and chronic physical, mental and disability-related health conditions among community-dwelling Medicare-Medicaid dually eligible enrollees. Archives of Community Medicine and Public Health.

[36] Shaughnessy TM, Parker FR, Hollenshead JH, Clottey EN, Rubin HW (2017). Contemporary Data and Trends in the Economic Costs of Mental Disabilities. Behavioral Sciences and the Law.

[37] Lindström E, Eberhard J, Neovius M, Levander S (2007). Costs of schizophrenia during 5 years. Acta psychiatrica Scandinavica Supplementum.

[38] Miles MB, Huberman AM, Saldana J (2018). Qualitative data analysis: a methods sourcebook.

[39] Voss CA, Tsikriktsis N, Frohlich M (2002). Case Research in Operations Management. International Journal of Operations & Production Management.

[40] World Health Organization. International Statistical Classification of Diseases and Related Health Problems. 2004.

[41] Flick U (2018). An Introduction to Qualitative Research.

[42] Miles MB, Huberman AM (1994). Qualitative data analysis: An expanded sourcebook.

[43] Patton MQ (2015). Qualitative Research & Evaluation Methods.

[44] Goetz JP, LeCompte MD (1984). Ethnography and qualitative design in educational research.

[45] Mays N, Pope C (2000). Assessing quality in qualitative research. BMJ.

[46] Eisenhardt KM (1989). Building Theories from Case Study Research. Academy of Management Review.

[47] Breen LJ (2007). The researcher “in the middle”: Negotiating the insider/outsider dichotomy. The Australian Community Psychologist.

[48] Pugh J, Mitchell M, Brooks F (2000). Insider/outsider partnerships in an ethnographic study of shared governance. Nursing Standard.

[49] Meredith J (1998). Building operations management theory through case and field research. Journal of Operations Management.

[50] McDonald R, Waring J, Harrison S (2006). Rules, safety and the narrativisation of identity: A hospital operating theatre case study. Sociology of Health and Illness.

[51] Christensen CM, Grossman JH, Hwang J (2009). The Innovator’s Prescription.

[52] Needham C. Best of both worlds: Comment on “(Re) making the procrustean bed? standardization and customization as competing logics in healthcare.” International Journal of Health Policy and Management [Internet]. 2018;7:356–8. Available from: 10.15171/ijhpm.2017.99 10.15171/ijhpm.2017.99 PMC5949228 29626405

[53] Porter ME, Lee TH (2013). The Strategy That Will Fix Health Care. Harvard Business Review.

[54] Dörbecker R, Böhmann T. The concept and effects of service modularity - A literature review. Proceedings of the Annual Hawaii International Conference on System Sciences. 2013;1357–66.

[55] de Mattos CS, Fettermann DC, Cauchick-Miguel PA. Service modularity: literature overview of concepts, effects, enablers, and methods. Service Industries Journal [Internet]. Taylor & Francis; 2019;0:1–22. Available from: 10.1080/02642069.2019.1572117

[56] Arman R, Liff R, Wikström E (2014). The hierarchization of competing logics in psychiatric care in Sweden. Scandinavian Journal of Management. Elsevier Ltd.

[57] O’Reilly CA, Tushman ML (2013). Organizational Ambidexterity: Past, Present and Future. Academy of Management Perspectives.

[58] Gibson CB, Birkinshaw J (2004). The antecedents, consequences, and mediating role of organizational ambidexterity. Academy of Management Journal.

[59] Havermans LA, den Hartog DN, Keegan A, Uhl-Bien M. Exploring the Role of Leadership in Enabling Contextual Ambidexterity. Human Resource Management [Internet]. 2015;54:s179–200. Available from: 10.1002/hrm.21764

[60] Act 2003:460 (amended SFS 2008:192). The act of ethical trail of research concerning humans. Ministry of Education and Research Sweden; 2008.

