# Supplementary material for: Value configurations for balancing standardization and customization in chronic care: a qualitative study
Source: BMC Health Serv Res. 2021 Aug 21;21:845. doi: 10.1186/s12913-021-06844-z (PMC8379884; doi:10.1186/s12913-021-06844-z)
Supplement: Supplementary file 1 — Additional file 1: Coding schemes for focus groups with illustrative examples [file 12913_2021_6844_MOESM1_ESM.docx]

# Additional file 1

# Coding schemes for focus groups with illustrative examples

Codes theoretically developed based on Fjeldstad et al. (2019) and further developed inductively in the coding process.

| Group | Code | Definition | Exemplary quotes |
| --- | --- | --- | --- |
| Value configurations | Chain | ﻿Linked repeatable, standardized processes | “If you come in as a patient, for example to the ER, there is a standardized process there, and from that process, the results of the assessments done, there is a result that one chooses how to handle.” (manager participating in focus group 1)  “What is striking is also that many of the chains that our care is built up by are not hard-linked, so that A must be followed by B, C, and D.” (developer participating in focus group 1)  “The process itself is thought of as a chain. We want to do a diagnostic investigation, a functional investigation and so on. But in practice it is more like a shop, because we look at the resources available…” (manager participating in focus group 2) |
|  | Shop | ﻿Highly customized responses to individual problems | “The idea is that the case manager does much of the standardized parts. And connected to that case manager is the interdisciplinary team with different expertise in the team to access. So there the patient can be the subject of interventions by others. But … the case manager, in clinical case management, possesses one part of the expertise.” (developer participating in focus group 1) |
|  | Network | ﻿Flexible interaction among people, places, and things. | “We shall work to help our patients together with them and, for example, have family education and social training and practice in communication with other persons. They might get phobia therapy so that they dare to go to the activity center. Very obvious network building. … The case manager is still the spider in the web, supporting the patient and providing the opportunity to have a more independent life.” (developer participating in focus group 2)  “Our patients, they are never really discharged. They are never cured. They just have a base level from which they get better or worse at times. So for us, the network is very important” (manager participating in focus group 1) |
|  | Parallel configurations | Several of the three value configurations coexisting more or less entangled. | “If we have a new patient who enters an investigation phase, then a chain model is applicable. At the same time, we want to get the teamwork started and then we are in a shop model. So new patients are in two models. And the main patient work at large is part of a shop. But then we have chronic patients where we have established contacts in the municipality and primary care. That, we sorted as a network, where our role becomes as marginalized as possible.” (manager participating in focus group 2)  “… we have a patient where we have worked intensively with [their care model] and the patient is stable. The network around the patient works. Then that is what is around the patient. But then the patient’s condition can get worse, and then the shop might get activated. If we even go to the ER, it becomes a chain.” (manager participating in focus group 1) |
|  | Appropriate level | Discussions about what level of abstraction at which to apply the lens of value configurations | “The higher up you go, the easier it is to see that we relate to a chain. The more you go into detail, the more you see the shop.” (developer participating in focus group 1) |
| Demands | Standardization | Focus on uniformity within or between processes or units, rather than on an individual’s needs or wishes. | “There are some parts of the process that are at least fairly standardized, but then, depending on the result, you individualize, and it becomes a shop. And then you might proceed to a chain and more standardized based on what you have concluded in the shop.” (manager participating in focus group 1) |
|  | Customization | Focus on the unique properties or needs of an individual patient or person rather than on process and organization. | “There is a [continuous] change between individual contact with the case manager, with the team, the resources of the team, and with acute care, to individualize the care and its content at the right level.” (developer participating in focus group 1) |
|  | Efficiency and resource management | Limitations and demands connected to economy, personnel, and management. | “Now when we have an increased inflow and we get more patients per case manager, we have to find a way of prioritizing to make best use of the time at hand. And if you look at the staff today, they are not able to think along those paths yet but are just overwhelmed with work. So it becomes my role as manager to try to guide them into a mindset that if we have not got enough time, then we have to put focus on the patients who need it the most.” (manager participating in focus group 2) |
| Drivers | External | Pressure and demands from external actors (e.g. government, patient organizations, and superior levels of the organization) | “For example, if we see that the Social Service does not take its responsibility, then we solve it for the patient. That is good for the patient, fur sure, but from an organizational point of view, it is not [good]. (manager participating in focus group 1)  “It is rather a survival strategy in some way, to move the resources from this group, who already have a lot in the society, to those who we have to concentrate the care and interventions to.” (manager participating in focus group 2) |
|  | Internal | Pressure and demands from internal actors (e.g. clinicians, developers or managers [can be themselves]) | *Developer:* “The staff might think mostly about somewhere that they have to do an active effort for us to measure, and then that is to register ‘KVÅ codes’. Other things are measured without their notice. But then, what we are going to research or inquire, that can be discussed. But that you [managers] can request, too.  *Manager:* Yes, and that may not be initiated yet, but soon. To look at measures that are closer to everyday operations and that feel important for that which they work with in everyday work.”  (participants in focus group 2) |
