# Supplementary material for: Value configurations for balancing standardization and customization in chronic care: a qualitative study
Source: BMC Health Serv Res. 2021 Aug 21;21:845. doi: 10.1186/s12913-021-06844-z (PMC8379884; doi:10.1186/s12913-021-06844-z)
Supplement: Supplementary file 2 — Additional file 2: Coding schemes for interviews with illustrative examples [file 12913_2021_6844_MOESM2_ESM.docx]

# Additional file 2

# Coding schemes for interviews with illustrative examples

Codes based on the analysis of the focus groups and theoretically derived from Fjeldstad et al. (2019) and Mannion and Exworthy (2017). Descriptions were used to strengthen intercoder reliability.

| Group | Code | Definition | Description | Exemplary quotes |
| --- | --- | --- | --- | --- |
| Value configurations | Chain | ﻿Linked repeatable, standardized processes | ﻿“It consists of linked repeatable, standardized treatment processes that professionals and patients use to produce the desired outcome.” | “The nearest that we have is probably the policlinic I believe. It is kind of an assembly line where the same 110 patients come during a four-week period … even if it is not entirely similar each time.” (I6) |
|  | Shop | ﻿Highly customized responses to individual problems | Shops in healthcare are “based on one-to-one patient–professional relationships where there is a predictable cycle of steps, including case acquisition, developing a diagnosis, selecting a customized treatment, and testing of the proposed solution.” | “The doctor first assesses the admission … and then it is handed over to the team where two persons are appointed – regardless of profession – who conduct a new patient-investigation and thereafter one takes in the activities needed. So if a psychologist investigation is needed, we involve one of the psychologists …” (I5) |
|  | Network | ﻿“Flexible interaction among people, places, and things.” | ﻿﻿﻿“A network is composed of nodes or ‘actors’ and the links that connect them.” “Network services provide the infrastructure to enable connections and exchange.” | “The interventions are still patient-bound, so especially in the treatment work it is around every individual patient that one can create a network that can take over [care and support activities].” (I4) |
|  | Parallel configurations | Several of the three value configurations coexisting more or less entangled. | Combined forms of organizational configurations for value creation in one unit or for one patient at a certain point in time. Can be truly mixed or distinguishable but tangled. | “What I would be sceptical of, is to see them as pure organizational configurations that you can select.” (I2)  ”It is much of a shop scenario, but we still have the network model as a target image to aim at. But we cannot do that – if we assume that we have a new patient that comes in, we still have some things we are imposed to do. Then, the further one comes in that process … the more one can incorporate the network.” (I4) |
| Demands | Standardization | Focus on uniformity within or between processes or units, rather than on individual’s needs or wishes. | Used for all efforts to standardize elements of care at various levels of abstraction, including design features, processes, performance, and terminology. | “I believe that, from a wider perspective, you have to have some form of standardization to see that the care is transparent and to find a lowest common denominator for what we can offer all patients.” (I4)  “The standard shows what we shall offer each patient and in that lies also that it shall be efficient and that we shall use the resources in the best possible way.” (I1)  “Looking at the standardized [elements] it is as if [the staff] gets stuck on that it shall be from A to Z and then they cannot use those standardized [elements] more customized and perhaps adapted to the patient’s needs and who the patient is.” (I1) |
|  | Customization | Focus on the unique properties or needs of an individual patient or person rather than on process and organization. | Used for all types of adaptation of care interventions or processes, either based on precision medicine, personalization, consumerism or self-care activities. | “And today I think it is so apparent with patient organizations and, I think, the entire discourse on participation and also thoughts about person-centered care, that there is a high pressure on us to invite to network meetings [and] for relatives to be asked to participate in meetings. And that is very good, but situations arise … where we struggle with other ideas: our professional judgement, routines for the unit, conflicts in a team …” (I2)  We have standardized care pathways but they do not really apply. I mean, we have them, but for our patients we cannot say that ‘this is what we are offering – take it or leave it’. But when the patient comes, we say, ‘What can we offer you?’ and so they may say what they might need and then we adapt in the extreme for them to get what they need.” (I3)  “it is not unusual among some young patients who are rather well-informed today and request some therapies, psychotherapy and so, and have an interest in talking more in-depth about themselves.” (I6) |
|  | Streamlining and scarce resources | Demands or restraints stemming from scarce resources, e.g. monetary or personnel. | Can be used both for top-down tasks or financial conditions and for experienced needs to cope with a situation of limited resources, such as difficulties recruiting enough competent employees. The common denominator is a demand to do more with the same or less resources of any kind. | “If we are to work by the models, we ought to work; if we shall offer all that we ought to, then we are too few employees. If you look at RACT [the main care model] and all models we work with, then one should not have more than 10-20-30 patients max. Nurses in my unit have 60 patients.” (I5) |
| Conflicting demands | Conflicting demands in general | Balancing and managing simultaneous internal and/or external demands, which can be seen as diverging or incompatible. | Used broadly for discussions about challenges connected to disparate requirements and conditions. Coded at group level for general discussions and at code level for combinations of specific pairs of demands. | “We are to develop towards some degree of standardization; we shall offer a battery of interventions. But we are also to adapt and work more individually with each patient in some parts. At the same time, we lose a lot of employees in the streamlining demands. So I see it … very clearly. And it is very contradictory if we are to fulfil it all.” (I5) |
|  | Standardization vs. efficiency | Management of parallel demands for standardization and improved efficiency or scarce resources. |  | “When there are demands for efficiency and standardization, then it is easier to say, ‘We have this way of working; this is what we are offering.’ It can be a limited number of visits, for example. Then there is an efficiency in that of course.” (I6)  “With requirements for increased efficiency, it is a contradictory demand to that we are obliged to conduct yearly physical examinations for all [patients]. That is what collides most for many, that it doesn’t add up when they see that there are other areas that one should work more with instead.” (I4) |
|  | Customization vs. efficiency | Management of parallel demands for customization and improved efficiency or scarce resources. |  | “Maybe we see that we should go in and offer individualized interventions but then this prioritization procedure comes in, that the largest possible effect for minimum input takes precedence over the high effect for high input.” (I4) |
|  | Standardization vs. customization | Management of parallel demands for standardization and customization. |  | “The problem is that we are to do all this. But on the other hand, it should all be person-centered and based on what the patient wants and not wants, and what needs he/she has, and what if there is a conflict there, that they want more or less than the standard says?” (I5)  “I think that individualization is easier [for the employees to accept] because that is something that many of them are passionate about. Then I think that standardization is perceived as something that is forced upon the operations … that it comes from politicians or the top management or our management group.” (I5) |
